# Microglia activity in the human basal ganglia is altered in alcohol use disorder and reversed with remission from alcohol

**DOI:** 10.1111/adb.13374

**Published:** 2024-02-08

**Authors:** Ameer Elena Rasool, Teri Furlong, Asheeta A. Prasad

**Affiliations:** ^1^ School of Medical Sciences, Faculty of Medicine and Health University of Sydney Sydney New South Wales Australia; ^2^ School of Medicine University of New South Sydney New South Wales Australia; ^3^ School of Psychology University of New South Sydney New South Wales Australia

**Keywords:** alcohol use disorder, astrocyte, microglia, post‐mortem human brain, remission

## Abstract

Alcohol use disorder (AUD) is characterized by cycles of abuse, withdrawal, and relapse. Neuroadaptations in the basal ganglia are observed in AUD; specifically in the putamen, globus pallidus (GP), and ventral pallidum (VP). These regions are associated with habit formation, drug‐seeking behaviors, and reward processing. While previous studies have shown the crucial role of glial cells in drug seeking, it remains unknown whether glial cells in the basal ganglia are altered in AUD. Glial cells in the putamen, GP, and VP were examined in human post‐mortem tissue of AUD and alcohol remission cases. Immunohistochemistry was performed to analyze cell count, staining intensity, and morphology of microglia and astrocytes, using markers Iba‐1 and GFAP. Morphological analysis revealed a significant decrease in microglia cell size and process retraction, indicating activation or a dystrophic microglia phenotype in individuals with AUD compared to controls. Microglia staining intensity was also higher in the GP and VP in AUD cases, whereas microglia staining intensity and cell size in remission cases were not different to control cases. In contrast, no astrocyte changes were observed in examined brain regions for both AUD and remission cases compared to controls. These results suggest alcohol exposure alters microglia, potentially contributing to dysfunctions in the basal ganglia that maintain addiction, and abstinence from alcohol may reverse microglia changes and associated dysfunctions. Overall, this study further characterizes AUD neuropathology and implicates microglia in the putamen, GP, and VP as a potential target for therapy.

## Graphical abstract

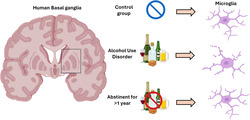

## 1 INTRODUCTION

Alcohol use disorder (AUD) is clinically characterised by the abuse of alcohol, dependence, drug‐seeking behaviours, withdrawal and vulnerability to relapse.^1^, ^2^ AUD is a chronically relapsing disorder, where around 60%–80% of individuals in remission return to their previous alcohol consumption habits.^3^ Alcohol seeking and relapse are modulated by reward centres in the brain, which centre on the ventral tegmental area and nucleus accumbens.^4^ In addition, the basal ganglia circuitry plays a complex role in drug addiction by regulating impulsivity, compulsivity, relapsing behaviour, habit formation and reward motivated reinforcement‐based learning.^5^, ^6^ It is well recognised that drugs of abuse, including alcohol, alter the structure and function of these regions to promote addictive behaviours and by extension increase vulnerability to relapse.^7^, ^8^ Hence, examination of basal ganglia structures, the putamen and globus pallidus (GP) and an associated nucleus, the ventral pallidum (VP), is important for understanding the maintenance of substance use disorders.

Initially, it was thought that the role of glial cells was to provide passive support to neurons.^9^ However, it is now evident that they also have an intrinsic role in regulating brain function such as forming and eliminating synapses and modulating neuroinflammation.^10^, ^11^ Specifically, microglia are recognised to be the innate immune cells of the central nervous system (CNS) where their functional state can be distinguished by their morphology. During the resting state, they possess ramified morphology, which is characterised by a small cell body and many long processes, and they release neurotrophic molecules.^9^, ^12^, ^13^ However, in the activated state, microglia appear in an amoeboid form, and release proinflammatory factors, which may have a neurotoxic effect in the CNS,^12^ and also increase immunoreactivity of microglial markers, such as ionised calcium binding adaptor protein‐1 (Iba‐1).^13^, ^14^ Astrocytes also have diverse functions by providing structural and metabolic support to neurons by regulating molecules exchanged in the blood brain barrier, and also promoting synaptic plasticity.^15^, ^16^, ^17^, ^18^ When responding to an insult, astrocytes undergo a process called astrogliosis, in which there is an increased tendency to proliferate, and an upregulation of glial fibrillary acidic protein (GFAP), a key cytoskeletal protein found on astrocytes.^19^, ^20^ Overall, neuropathological studies have shown that expression of Iba‐1 and GFAP can be used as measures of microglia and astrocyte activation.^15^, ^21^, ^22^

Drugs of abuse can interact with glial cells to induce changes in their activity, consequently altering neuroimmune and neuronal functioning, which is thought to lead to impaired function of critical brain regions that promote addictive behaviours.^2^, ^10^ For instance, analysis of human post‐mortem tissue from individuals with AUD found increased expression of markers of microglia in the cingulate cortex, ventral tegmental area and midbrain.^14^ Additionally, in rodents, prolonged, intermittent administration of alcohol led to increased activation of microglia, in the hippocampus and entorhinal cortex, which was accompanied with increased presence of proinflammatory cytokines, neurodegeneration and cognitive deficits.^13^ Importantly, abstinence from alcohol is associated with an increase in resting microglia compared to when alcohol is continued to be consumed intermittently, along with dendritic spine restoration, and improvements in cognitive deficits and neurodegeneration.^13^ In addition, astrocytes have been shown to be altered by alcohol exposure. For example, prolonged alcohol consumption in rats has been shown to decrease astrocyte numbers as indicated by GFAP immunoreactivity in the hippocampus.^23^, ^24^, ^25^ Further, prolonged alcohol exposure has been shown to prevent the activation of cultured astrocytes taken from human brain tissue.^26^ This inhibitory effect of alcohol on astrocytes impairs astrocyte functions, such as neurotransmission and synapse formation, which may lead to dysfunctions in normal neuronal signalling that promote alcohol seeking behaviours.^27^ Hence, alcohol has been shown to alter both microglia and astrocytes in the cerebral cortex and other brain regions such as hippocampus but has yet to be investigated in basal ganglia structures.

The current study examined Iba‐1 and GFAP expression as measures of microglia and astrocyte changes in post‐mortem tissue from AUD brains. Remission cases were also examined in order to determine the impact of abstinence of alcohol on brain recovery. The putamen, GP and VP were examined due to their important role in addiction processes, and the impact of alcohol on glia in these regions has yet to be examined. Here, we show that microglia morphology is altered in AUD brains compared to controls and remission groups. However, no change in astrocyte marker levels were detected suggesting specific glial cell recruitment in AUD.

## 2 METHODS

### 2.1 Tissue samples and cases

Formalin fixed and paraffin embedded 14‐μm coronal sections of human brain tissue were obtained from the NSW Brain Tissue Research Centre (BTRC). Ethics for this project was approved by the Human Research Ethics Committee at UNSW (HC200756). Clinical history per case was assessed by a psychiatric clinician to accurately characterise and ensure cases meet DSM‐IV criteria for AUD.^28^ Three sample groups were used for this study, control, AUD, and remission as described in Harper et al.^29^ Individuals who had consumed more than ~80 g of alcohol per day met the criteria for the AUD group, whereas individuals who had consumed less than ~20 g met the criteria for the control group. The remission group included individuals who have previously had a diagnosis of AUD but discontinued drinking and stayed abstinent for 1 or more years. Groups were matched for sex and age. A total of 43 cases were initially considered for inclusion in the study including 17 AUD, 17 control and nine remission cases (see Table 1).

**TABLE 1:** Case characteristics of all subjects.

Patient ID	Group	Age	Sex	PMI	Brain pH	Clinical COD	Years abstinent
CO2	Control	51	M	35	7.00	Cardiac	‐
C22	Control	64	M	29	6.55	Cardiac	‐
C32	Control	61	M	30	6.69	Cardiac	‐
C04	Control	66	M	32	6.66	Cardiac	‐
C14	Control	53	M	26	6.36	Cardiac	‐
C34	Control	52	M	28	6.28	Cardiac	‐
C06	Control	62	M	30	6.67	Cardiac	‐
C16	Control	49	M	22	6.88	Cardiac	‐
C26	Control	58	M	68	6.82	Cardiac	‐
C09	Control	54	F	23	6.17	Toxicity	‐
C19	Control	45	F	29.5	6.78	Cardiac	‐
C29	Control	76	M	18	6.04	Renal	‐
C38	Control	67	M	64	5.60	‐	‐
C12^a^	Control	58	M	39	6.49	Cardiac	‐
C24^a^	Control	40	M	59	6.93	Vascular	‐
C36^a^	Control	29	F	40	6.83	Cardiac	‐
C39^a^	Control	77	F	‐	6.17	Metabolic	‐
A13	AUD	32	F	62	6.41	Toxicity	‐
A23	AUD	62	M	30.5	6.79	Cardiac/Respiratory	‐
A05	AUD	61	M	52	6.63	Cardiac	‐
A25	AUD	51	M	51.5	6.80	Hepatic	‐
A07	AUD	71	M	39	6.80	Cardiac	‐
A17	AUD	55	M	24	6.43	Cardiac	‐
A27	AUD	56	M	39.5	6.34	Hepatic/Blood loss	‐
A37	AUD	51	F	37	6.95	Toxicity	‐
A08	AUD	82	F	28	6.40	Hepatic	‐
A18	AUD	53	M	61	6.79	Cardiovascular	‐
A28	AUD	47	F	69	6.24	‐	‐
A42	AUD	63	M	47	6.76	Cardiac	‐
A43	AUD	51	M	43	7.05	Cardiac/Hepatic	‐
A03^a^	AUD	63	M	28	6.89	Respiratory	‐
A33^a^	AUD	50	M	34.5	6.93	Respiratory/Toxicity	‐
A15^a^	AUD	40	M	50.5	6.83	Alcohol and Obesity	‐
A35^a^	AUD	48	M	22	6.18	Toxicity	‐
R40^b^	Remission	84	M	28.5	5.92	COPD	5
R41	Remission	90	M	22.5	5.66	Infection	32
R20	Remission	69	M	48	6.26	Cardiac	19
R01	Remission	44	M	15	6.48	Cardiac	14
R11	Remission	80	M	28	6.25	Cardiac/Respiratory	20
R21	Remission	71	M	9.5	6.57	Cardiac	1
R31	Remission	75	F	9	6.00	Hepatic	5
R10^b^	Remission	26	F	40	6.67	Respiratory	1
R30^b^	Remission	56	F	47	6.72	Toxicity	3

^a^ Cases that were excluded from study.

^b^ Cases with comorbidities: R40 comorbidity with Parkinson's disease; R10 comorbidity with schizophrenia; R30 comorbidity with benzodiazepine dependence.

### 2.2 Immunohistochemistry

The immunohistochemistry process was adapted from the manufacturer's (Leica Biosystems, REF# RE7140‐K) instructions of the Novolink polymer detection system (see previous work).^30^ Paraffin‐embedded brain sections were dewaxed in xylene, followed by incubation a series of decreasing ethanol concentrations (100%, 95%, 70%; 3 min each). Samples were treated for antigen retrieval in 0.1 M citrate buffer (pH 6) and cooled (30 min) before washing in milli Q water. Slides to be stained with Iba‐1 were then emersed in 0.2% Tween‐20 (15 min) to prevent non‐specific binding of the primary antibody. Slides were then incubated (5 min) with a peroxidase block (3%–4% hydrogen peroxide) to prevent endogenous peroxidase activity. Next, slides were incubated (15 min) in a protein block (0.4% Casein in phosphate‐buffered saline, with stabilizers, surfactant, and 0.2% Bronidox L as a preservative) to prevent nonspecific binding of antibodies. Primary antibodies, Iba‐1 (1:700; Anti‐Iba‐1, [019–19,741], rabbit polyclonal; Wako Pure Chemical Industries, Japan) and GFAP (1:1000; anti‐GFAP, AB7260, rabbit polyclonal; Abcam), were diluted with 0.1 M tris‐buffered saline (pH 7.5) and applied to samples for overnight incubation (anti‐GFAP was incubated at 4°C and anti‐Iba‐1 at room temperature). Next, the sections were incubated (30 min) in a post‐primary block (Rabbit anti‐mouse IgG in 10% animal serum in tris‐buffered saline/0.1% ProClin™ 950) and then a Novolink polymer (anti‐rabbit Poly‐HRP‐IgG containing 10% animal serum in tris‐buffered saline/0.1% ProClin™ 950) to detect and visualize the antibodies. Finally, a Novolink 3,3′‐diaminobenzidine (DAB) chromogen solution was used to react with peroxidase to create a brown precipitate at the antigen site (incubated for 5 min) and hematoxylin was added (incubated for 3 min) to counterstain cell nuclei. Slides were washed with reverse osmosis water, then dehydrated in increasing concentrations of ethanol, followed by xylene and coverslipped with Entellan (IM022; Prositech; Kiriwin, Australia).

### 2.3 Image acquisition and region identification

**FIGURE 1:**
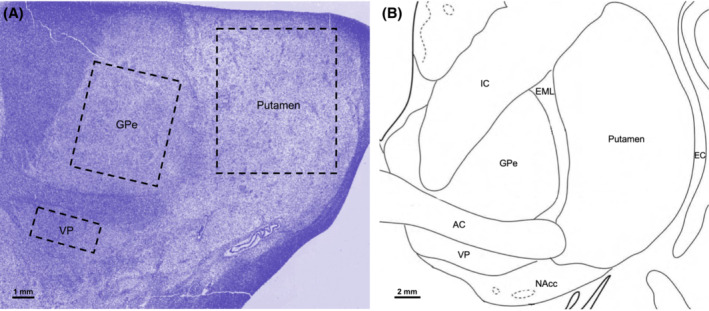
*Representative images of coronal section and ROI analysed.* (A) *Haematoxylin‐stained section of* regions of interest (*ROIs*)*.* Representative image of rectangular ROIs analysed in the putamen, external globus pallidus (GPe) and ventral pallidum (VP) (black dotted line). (B) *Image of coronal section corresponding to the coronal level of the section in (A**)***. Modified from the Stereotactic Atlas of the Human Thalamus and Basal Ganglia.^32^ All structures of interest can be seen in addition to landmark structures used to identify boundaries, such as the internal capsule (IC), external medullary lamina (EML), external capsule (EC), anterior commissure (AC) and nucleus accumbens (NAcc)

Images of whole slide were captured by the Aperio Scanscope at 20x magnification and a blind analysis of Iba‐1 and GFAP in brain sections was performed using Qupath v.0.3.2.^31^ A single brain section was analysed per case for Iba‐1 only or GFAP only. Representative images of Iba‐1^+^ cells (Figure 5) were taken at 40X using the Nikon Ni‐E Basic Fluorescence Widefield microscope. Anatomical boundaries of the putamen, GP, and VP were delineated using landmark structures, such as the anterior commissure and internal capsule, as determined by the Stereotactic Atlas of the Human Thalamus and Basal Ganglia.^32^ Additionally, the haematoxylin stain was used to visualise the accurate boundaries of each region, which were annotated using the polygon drawing tool on Qupath. A single rectangular region of interest (ROI) in each structure was analysed (see Figure 1) The coronal level of interest was a distance of 25.0 to 28.5 mm anterior to the posterior commissure. At this level there was the complete presence of the anterior commissure, and thus all regions of interest were present.^32^ Cases without sections at this level (i.e., with incomplete or absent anterior commissure) were excluded from the analysis (see Section 3). The GPe was defined as the area that lies dorsal to the anterior commissure, lateral to the internal capsule and medial to the putamen, as identified in Pauli, Nili,^33^ with the external medullary lamina separating the GPe and the putamen. At this level, the internal GP was not present. The putamen's lateral boundary was identified as the external capsule, and due to its poorly defined ventral limit,^33^ the ventral boundary of the putamen was considered to be in line with the dorsal aspects of the anterior commissure. The VP was identified as the crescent‐like structure in the pallidal region that lies directly ventral to the anterior commissure, and at this rostral level, the VP lies adjacent to the nucleus accumbens.^34^

### 2.4 Quantification

Qupath software^31^ was used to quantify all measures, which included cell density, staining intensity, cell size, cell body area and process area. All quantification was performed with the experimenter blind to group. Cell Density was measured using the ‘Positive Cell Detection’ function in Qupath to analyse the entire ROI for each brain structure (defined above), which determined the number of cells present. A manual count of cells performed by the experimenter, blind to group, to confirm accuracy of selected thresholds for each case which was determined, with correlation of above 0.90. All cell counts expressed as number of cells/mm^2^. Staining intensity was extracted from the positive pixels in the entire ROI using the ‘Positive Pixel Count’ function in Qupath and expressed as a value of optical density of DAB in each pixel. Thresholds for staining intensity were manually selected and adjusted for each ROI to ensure all cell bodies and processes were detected. For measures of cell size, the ‘Positive Pixel Count’ function in Qupath was used to determine the area of positive pixels which was then corrected by the number of cells in the ROI, in order to calculate the average size of a cell. Microglia cell bodies were detected if they had a minimum area of 30 μm^2^. The area of the cell body was determined using the ‘Positive Cell Detection’ function (as described above) and subtracted from the average area of each cell in each ROI to give the average area of the cell's processes (see Figure S1). All measurements of area were expressed as μm^2^.

### 2.5 Statistical analysis

All statistical analysis was performed using IBM SPSS Statistics software (Released 2019. Version 26.0. Armonk, NY: IBM Corp). Figures were created using Graphpad Prism 9 (version 9.4.1 for Mac OS X, Graphpad Software, San Diego, California, USA). All data was averaged according to three groups: control, AUD, and remission groups. For all case characteristic data and cell quantification data, a non‐parametric Kruskal–Wallis test was performed to determine differences in sex, age, post‐mortem interval (PMI), and brain pH and compare the three groups. When results were significant, a post hoc analysis using Dunn's test with Bonferroni correction for multiple comparisons was then performed to determine any differences between each group. Data was reported as mean ± SEM. Results were considered significant if *p* < 0.05.

## 3 RESULTS

### 3.1 Analysis of case characteristics

Eight cases were excluded from all analysis as the brain sections were not at the correct coronal level and did not have all structures of interest present, or because of a lack of information for PMI available (see Table 1). Cases from the remission group with comorbidities or low pH values were not excluded due to limited tissue availability, thus examination of these cases was needed to ensure sufficient sample size. Final group numbers were *n* = 13 for control, *n* = 13 for AUD and *n* = 9 for remission. Mean values for case demographics are summarised in Table 2. A Kruskal–Wallis analysis confirmed no significant difference between controls, AUD group, and remission group for sex (H[2] = 1.140, *p* = 0.566), age (H[2] = 3.023, *p* = 0.221) and brain pH (H[2] = 5.376, *p* = 0.068). However, there was a significant between groups for PMI (H[2] = 7.453, *p* = 0.024). Post hoc analysis found there was no significant difference in PMI between control and AUD group (*p* = 0.119) or between control and remission group (*p* = 1.000), but a significant difference between AUD and remission group (*p* = 0.034).

**TABLE 2:** Summary of patient demographics and mean values of case characteristics.

	Control	AUD	Remission
**Sex**	*n* = 13 (11 M, 2 F)	*n* = 13 (9 M, 4 F)	*n* = 6 M, 3 F
**Age**	58.308 ± 2.408	56.539 ± 3.349	66.111 ± 6.867
**PMI**	33.423 ± 4.202	44.885 ± 3.832^a^	27.500 ± 5.007
**Brain pH**	6.500 ± 0.109	6.645 ± 0.071	6.281 ± 0.122

^a^ Cases that were excluded from study.

### 3.2 Analysis of GFAP immunoreactivity

**FIGURE 2:**
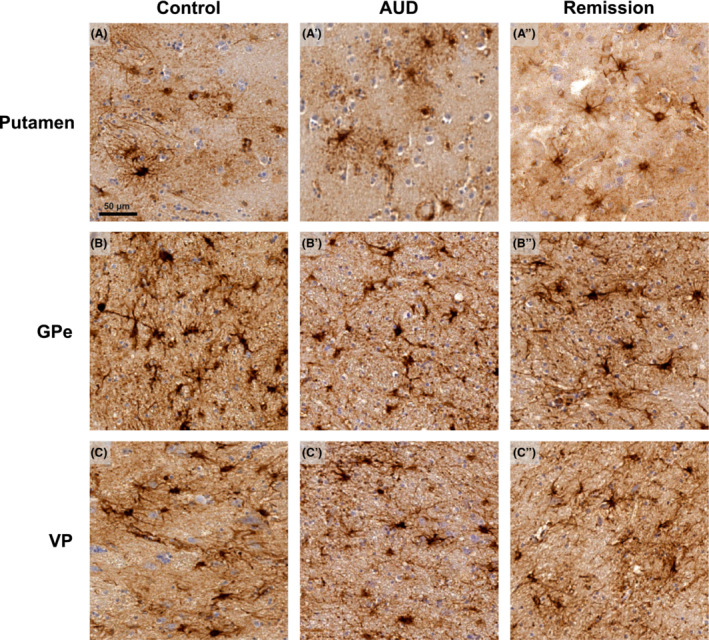
*Astrocytes in human post‐mortem tissue. (*A‐C″). *Representative images of GFAP* ^*+*^ *cells in the putamen, GPe, and VP of control, AUD and remission groups.* Cell number, staining intensity and cell area appear visually similar across all three groups in all structures

**FIGURE 3:**
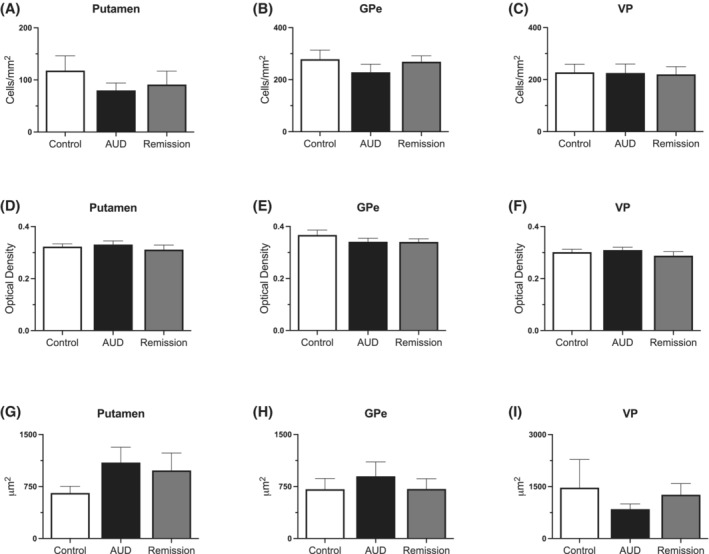
*GFAP immunoreactivity.* (A–C) *Number of GFAP* ^*+*^ *cells.* Quantification of GFAP^+^ cell density, expressed as cells/mm^2^, show no significant difference in the number of cells across all groups in the putamen, GPe and VP. (D–F) *Staining intensity of GFAP* ^*+*^ *cells*. Quantification of GFAP^+^ staining intensity, expressed as a value of optical density, show no significant difference in staining intensity between control, AUD, and remission groups in the putamen, GPe, and VP. (G–I) *Average size of GFAP* ^*+*^ *cells.* Quantification of GFAP^+^ cell area, expressed as μm^2^, show no significant difference in the average area of a cell between control, AUD and remission groups in the putamen, GPe, and VP. Results are shown as mean ± SEM. Groups are control (*n* = 13), AUD (*n* = 13) and remission (*n* = 9)

Analysis of astrocytes included the number of GFAP^+^ cells and staining intensity in the putamen, GPe and VP. Representative images are shown in Figure 2 where the number of GFAP^+^ cells and staining intensity appears similar across all groups in all brain regions examined. A Kruskal–Wallis test confirmed no significant difference in the number of GFAP^+^ cells between the control, AUD and remission group in the putamen (H[2] = 0.421, p = 0.810), GPe (H[2] = 0.913, *p* = 0.633) and VP (H[2] = 0.065, *p* = 0.968) (see Figure 3A–C). Thus, indicating that there was no difference in the number of astrocytes across groups. In addition, analysis of staining intensity, expressed as a value of optical density, was used to measure astrocyte activity. No significant difference in GFAP staining intensity was detected between all three groups within the putamen (H[2] = 1.119, *p* = 0.571), GPe (H[2] = 1.144, *p* = 0.564) and VP (H[2] = 2.233, *p* = 0.327) (see Figure 3D–F). Moreover, it was found that there was no significant difference in cell size within control, AUD and remission in the putamen (H[2] = 1.922, *p* = 0.383), GPe (H[2] = 0.310, *p* = 0.856) and VP (H[2] = 1.429, *p* = 0.490), (see Figure 3G–I). Together these findings demonstrate no change in immunoreactivity of GFAP in AUD or remission groups.

### 3.3 Analysis of Iba‐1 immunoreactivity

**FIGURE 4:**
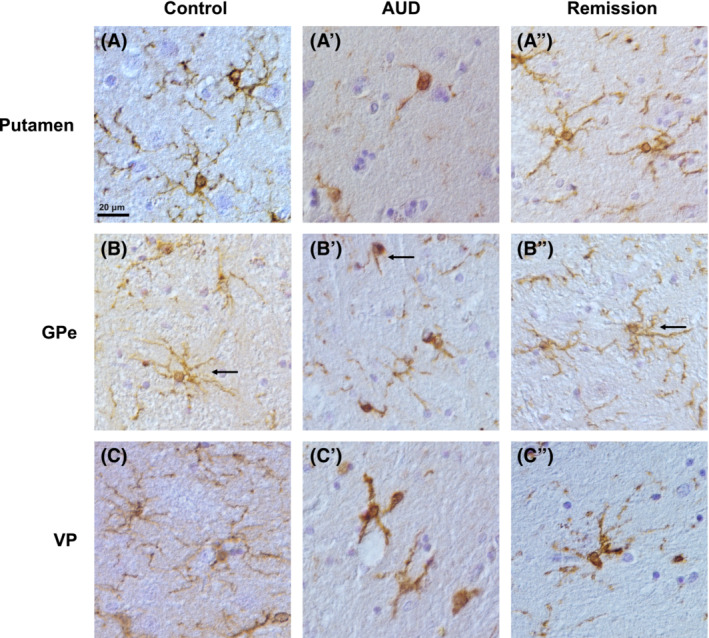
*Microglia in human post‐mortem tissue.* (A–C**″**) *Representative images of Iba‐1* ^*+*^ *cells in the putamen, GPe and VP of control, AUD and remission groups.* Differences in staining intensity between each group is visible, with Iba‐1^+^ cells in the AUD group showing a darker staining compared to the control and remission groups for all regions examined. Differences in morphology of microglia is also visible, with cells in the control group appearing to possess a more ramified morphology and an overall larger cell area, cells in the AUD group having less ramified processes, thus smaller cell areas, and cells in the remission group appearing similar to the control group (see insert, top right of each column)

To investigate changes in number and activity of microglia associated with AUD and abstinence from alcohol, Iba‐1^+^ cell density, staining intensity and cell size were examined in the putamen, GPe and VP. Representative images are shown in Figure 4 where the number of cell bodies does not appear to be different across groups but changes in staining intensity and cell size and processes are evident for the AUD group compared to the other two groups. Specifically, microglia in the control group appeared to be lightly stained, and possessed a ramified morphology, as cell bodies were surrounded by multiple long processes in all brain regions shown. In contrast, microglia in the AUD group appeared to have darker cell bodies with less and shorter processes than the control group, whereas microglia in the remission group appeared to have similar staining intensity of cells, as well as similar cell size and number and length of processes, as the control group.

**FIGURE 5:**
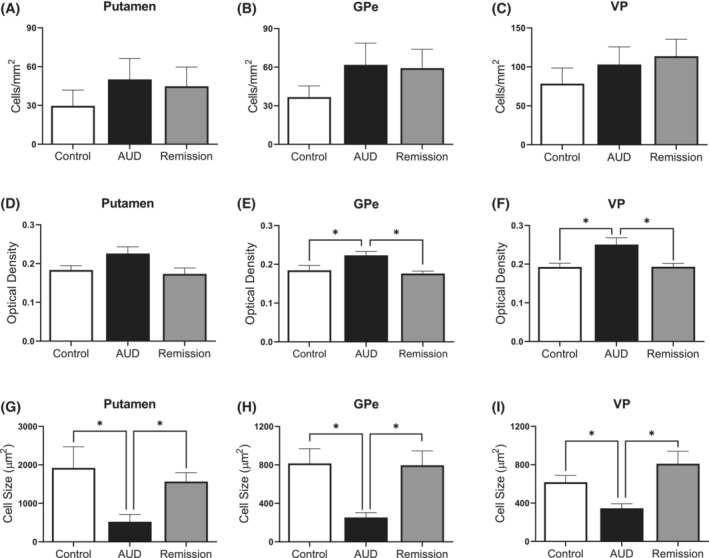
*Iba‐1 immunoreactivity.* (A–C) *Number of Iba‐1* ^*+*^ *cells.* Quantification of Iba‐1^+^ cell density. Expressed as a value of cells/mm^2^ show no significant difference in the number of cells across all groups in the putamen, GPe and VP. (D–F) *Staining intensity of Iba‐1* ^*+*^ *cells.* Quantification of Iba‐1^+^ staining intensity expressed as a value of optical density shows that staining intensity in the AUD group was significantly greater than both the control and remission group in the putamen, GPe and VP. There is no significant difference in staining intensity between the control and remission group across all three structures. (G–I) *Average size of Iba‐1* ^*+*^ *cell.* Quantification of Iba‐1^+^ cell area, expressed as μm^2^, shows that the average cell size in the AUD group was significantly lower than both the control and remission groups in the putamen, GPe, and VP. There was no significant difference in the average cell size between the control and remission groups in all examined structures. Results are shown as mean ± SEM. For significant results *p* < 0.05 indicated by *. Groups are controlle (*n* = 13), AUD (*n* = 13) and remission (*n* = 9)

For the number of Iba‐1^+^ cells a Kruskal–Wallis test revealed there was no significant difference for any of the groups examined for the putamen (H[2] = 2.699, *p* = 0.259), GPe (H[2] = 1.975, *p* = 0.372) or VP (H[2] = 2.130, *p* = 0.345). Thus, indicating that numbers of microglia in each structure did not change across groups (see Figure 5A–C). Further analysis of microglia activity was performed where optical density was used to measure staining intensity, as shown in Figure 5D–F. A Kruskal–Wallis test revealed significant differences between groups in staining intensity of Iba‐1 in the putamen (H[2] = 6.281, *p* = 0.043), GPe (H[2] = 9.242, *p* = 0.010) and VP (H[2] = 10.016, *p* = 0.007) (see Figure 5D–F). Post hoc analysis in the putamen, however, revealed that staining intensity was not significantly different for the AUD group compared to both the control (*p* = 0.146) and remission (*p* = 0.069) groups, or between the control and remission groups (*p* = 1.000). Post hoc analysis for the GPe revealed that staining intensity was greater in the AUD group compared to both the control group (*p* = 0.041) and the remission group (*p* = 0.021), while the control and remission groups did not differ to each other (*p* = 1.00). Finally, post hoc analysis for the VP also revealed greater intensity of staining in the AUD group compared to both the control (*p* = 0.046) and remission (*p* = 0.011) groups, but not between the remission and control groups (*p* = 1.000). Thus, there was an upregulation of Iba‐1 in the AUD group compared to the control in the GPe and VP, whereas the remission group returned to control levels of Iba‐1 immunoreactivity.

**FIGURE 6:**
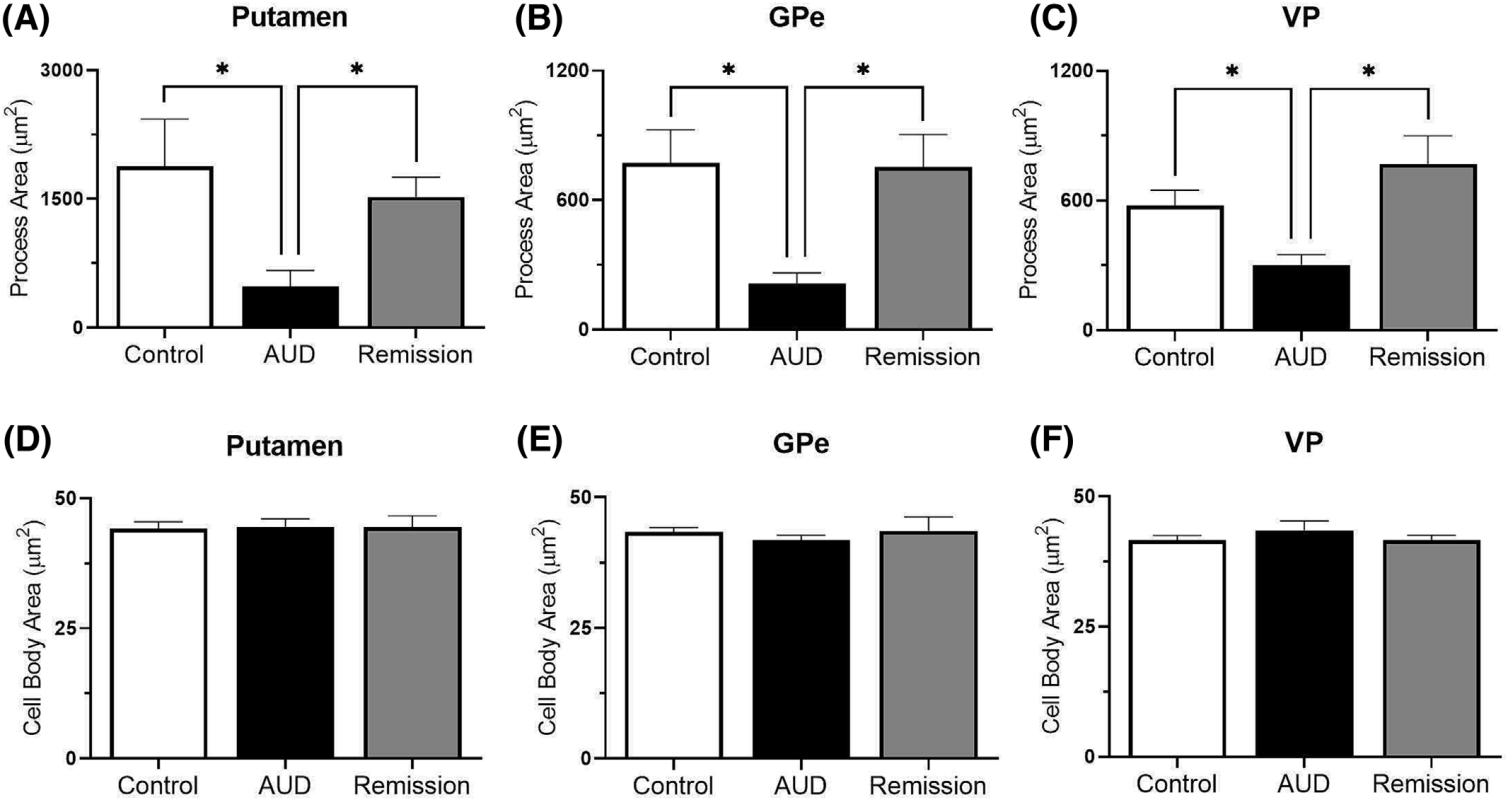
Iba‐1^+^ area of processes and cell body. (A–C) *Area of processes of Iba‐1* ^*+*^ *cells.* Quantification of Iba‐1^+^ cell processes, expressed as μm^2^, shows that average area of processes in the AUD group is significantly lower compared to the control and remission group, which have similar process areas in the putamen, GPe and VP. (D–F) *Area of cell bodies of Iba‐1* ^*+*^ *cells.* Quantification of cell bodies of Iba‐1^+^ cells, expressed as μm^2^, show no significant difference in the area of cell bodies across all groups in the putamen GPe, and VP

Further analysis of microglia morphology was performed to elucidate the potential activation state of microglia by measuring cell size, process area, and cell body area. Analysis of cell size showed significant differences in the putamen (H[2] = 12.681, *p* = 0.002), GPe (H[2] = 14.492, *p* = 0.001) and VP (H[2] = 13.262, *p* = 0.001) (see Figure 5G–I). Post hoc analysis for all regions confirmed that cell area was lower for the AUD group compared to the control group (*p* = 0.007, *p* = 0.004 and *p* = 0.033, for putamen, GP and VP, respectively) as well as compared to the remission group (*p* = 0.007, *p* = 0.003 and *p* = 0.002, for putamen, GP and VP, respectively), but not for the control group compared to the remission group (*p* = 1.00, *p* = 1.00 and *p* = 0.724, for putamen, GP and VP, respectively). Similarly, the area of processes differed significantly between groups for the putamen (H[2] = 12.681, *p* = (0.002), GPe (H[2] = 13.974, *p* = 0.001), and VP (H[2] = 13.068, *p* = 0.001) (see Figure 6A–C). Post hoc analysis indicated that process area for all regions was significantly lower in the AUD group compared to the control group (*p* = 0.007, *p* = 0.05 and *p* = 0.037, for putamen, GP and VP, respectively) and the remission group (*p* = 0.007, *p* = 0.004 and *p* = 0.002, for putamen, GP and VP, respectively), but not for the control group compared to the remission group (*p* = 1.00, *p* = 0.01 and *p* = 0.704, for putamen, GP and VP, respectively) (see Figure 6A–C). Finally, analysis of cell body area revealed no significant difference across all groups in all three brain regions: the putamen (H[2] = 0.099, *p* = 0.951), GPe (H[2] = 3.161, *p* = 0.206) and VP (H[2] = 1.125, *p* = 0.570) (see Figure 6D–F). Thus, although there was no significant difference in the number of microglia, changes in cell size and process area indicate that microglia may be undergoing morphological alterations in AUD cases compared to control and remission cases.

## 4 DISCUSSION

The current study examined changes in microglia and astrocytes associated with AUD (using Iba‐1 and GFAP immunostaining), within three brain regions that have been implicated in addiction‐related behaviours: the putamen, GP and VP. It was found that while the number of Iba‐1^+^ cells did not change across groups, the staining intensity of Iba‐1^+^ cells was greater for the AUD group in the GPe and VP compared to control and remission cases. This led to further examination factors impacting the change in intensity. The cell size and process area of microglia were also lower for all regions examined in the AUD group compared to the other two groups. These findings suggest that prolonged alcohol use induces microglia activation, indicated by morphological changes in microglia. Moreover, levels of staining intensity of Iba‐1^+^ and cell and process area in the remission group were similar to the controls for all regions examined, thus suggesting recovery of microglia during remission. Overall, these findings demonstrate that alterations to microglia are part of AUD cellular pathology. In contrast, the number and staining intensity of GFAP^+^ cells did not change across groups, indicating that activity of astrocytes may not change within the putamen, GPe and VP in AUD. To our knowledge, this is the first study to characterise changes in glial cells within the putamen, GPe and VP in AUD and after at least 1 year of abstinence in human post‐mortem tissue and is thus an important step towards fully characterising pathology changes in AUD. The putamen, GPe and VP are responsible for habit formation, motivation, and reward, all of which drive alcohol‐seeking behaviours that are characteristic of AUD.^8^, ^35^ Thus, activation of microglia within these structures may cause dysfunctions in neuronal communication to promote and maintain AUD. Therefore, the findings of the current study further implicate the examined brain regions, and in particular microglia within these regions, as a potential target for treatment.

The current study indicates that while the number of microglia does not change in AUD, staining intensity increased and morphology was altered, as demonstrated by decreased cell size. The decrease in cell size is likely due to retraction of cell processes, given that there was a reduction in the area of the processes but not in the area of the cell bodies (as seen in Figures 5G–I and 6A–C). The increase in Iba‐1 staining is indicative of activation of microglia while the lack of enlarged cell body indicates that the microglia did not reach a fully activated (amoeboid) state.^12^, ^13^, ^36^ Furthermore, the reduction in microglia processes that was observed is recognised to occur when activated microglia retract with prolonged brain insult.^37^ Increased density of Iba‐1 staining has also been reported in the cingulate cortex in human AUD cases, which was interpreted as increased activation of microglia.^14^ In contrast, there was no change in Iba‐1 staining in the amygdala, which indicates that microglia activation is brain‐region specific.^14^ Therefore, the current study extends on these prior findings and suggests that in addition to the cingulate cortex, the putamen, GPe and VP are also brain regions of high vulnerability to alcohol‐induced inflammatory changes. While it is unknown why microglia in only some brain regions respond to alcohol, it is known that microglia are heterogenous, and are recognised to differ in their density, molecular markers, and function across different brain regions.^38^, ^39^ These differences are likely to contribute to the varied sensitivity of microglia across brain regions that has been demonstrated to a range of physiological conditions such as ageing, stress and lipopolysaccharide insult,^39^ and thus may also be responsible for regional responsivity to alcohol. Furthermore, similar to the current findings, studies in Alzheimer's disease demonstrate that the number of microglia are unchanged, but the expression of Iba‐1 is increased.^40^ Together these studies suggest that existing microglia upregulate their Iba‐1 expression in response to altered physiological conditions, rather than recruiting more microglia to susceptible brain regions.

It has been proposed that the pro‐inflammatory effects of microglia contribute to the degeneration of neurons associated with alcohol exposure.^36^, ^41^, ^42^ Thus, in order to fully understand the role of microglia in AUD it would be necessary to determine whether the altered state of microglia in the current study is associated with neuroprotection and the release of trophic and anti‐inflammatory factors, or whether microglia are contributing to neurotoxic effects in the brain by producing cytotoxic factors.^36^, ^43^ This could be achieved by examining specific neuroprotective and neurotoxic markers associated with these functional states, as Iba‐1 is found in ramified, activated, amoeboid, and dystrophic microglia, it cannot distinguish whether microglia are displaying the pro‐ or anti‐inflammatory phenotype.^43^, ^44^, ^45^ Therefore, further investigation of different microglial phenotypes present using markers specific to different morphologies, such as CD68, MHCII and ferritin is needed.^45^ Of note, prior studies in rodents indicate that prolonged exposure to alcohol, as would have occurred in the AUD group, results in a pro‐inflammatory microglial phenotype. Specifically, intermittent, repeated binge, and long‐term exposure to alcohol in rodents promotes pro‐inflammatory factors that contribute to neuron death.^13^, ^43^, ^46^, ^47^ Further, the reduced area of processes of microglia seen in the current study, is also associated with pro‐inflammatory responses.^37^ However, this would be needed to be confirmed for AUD as alcohol exposure has been shown to increase both pro‐ and anti‐ inflammatory microglia.^43^ Finally, it would also be of interest to determine how alcohol interacts with microglia. Several key targets have been identified including toll‐like receptors, various microRNA and cytokines, including high‐mobility group box 1 and MAPK signalling pathways,^41^, ^42^, ^48^ as well as adenosine‐receptors which are thought to be involved in reducing microglia processes as occurred in the current study.^37^ Another possibility is that microglia present in the AUD group exhibit a dystrophic morphology, which is a novel state, thought to be senescent, that is more prevalent in older populations.^45^, ^49^ Dystrophic microglia display morphological abnormalities such as de‐ramified or fragmented processes and spheroid formation^45^, ^49^ and in the current study microglia processes were found to be significantly reduced in the AUD group. A rodent model of alcohol dependence found that, while there was a loss of Iba‐1^+^ cells, there was a significant number of dystrophic microglia in the remaining cells in the hippocampus.^50^ This suggests that alcohol may be toxic to microglia and potentially limit their ability to provide support to neurons.^50^ These dystrophic microglia have been associated with functional loss, such as diminished inflammatory and phagocytic responses, and decreased ability to release neurotrophic factors^45^, ^50^ and hence would also suggest dysfunction in structures in the current study for the AUD group regardless of whether microglia are activated or dystrophic. Moreover, aging may have affected the morphology of microglia. A study comparing microglia in non‐diseased post‐mortem tissue from a 38 and 68‐year‐old brain found that while dystrophic microglia were rare in the younger subject, they were widespread in the older subject.^49^ Thus, as tissue used in this study are mostly from elderly subjects (on average above the age of 55), it is possible that microglia present may have been affected by ageing and exhibit the dystrophic phenotype, but this would have impacted all groups equally given that there was no difference in the average age of each group.

In the current study, there was a reduction of active microglia in the remission group compared to the AUD groups, as indicated by Iba‐1 staining intensity and cell size, which did not differ from controls. Changes in Iba‐1 staining intensity and cell size suggests that abstinence from alcohol may result in mitigation of microglia activation and the amelioration of neuroinflammation in the putamen, GPe and VP after at least 1 year of abstinence. A rodent study similarly showed recovery of microglia morphology following abstinence from alcohol which was accompanied by improved cognitive function^13^ Importantly, it has been shown that the persistence of neuroinflammation after abstinence from alcohol predicts relapse in rats^3^ and suppression of microglia with minocycline in mice reduces voluntary alcohol intake.^51^ This suggests that neuroinflammation may promote alcohol‐seeking behaviours and hence the reduction in microglia activation in the current study for the remission group may protect against relapse. Moreover, as microglia are known to play a role in neuroplasticity by synaptic pruning and remodelling, dysfunction of microglia may have an adverse impact on activity of neuronal circuits and associated brain functions.^50^, ^52^ Therefore, the observed return of Iba‐1 staining intensity and cell size to levels similar to healthy controls, suggests normal microglia function may be restored and offers hope for recovery of damage induced by chronic alcohol consumption. However, it should be noted that the PMI of the remission group differed to the AUD group. Thus, it may be the case that differences in Iba‐1 staining may be due to PMI rather than from remission from alcohol (although PMI did not differ between control group and the remission group). Of note, it has been suggested that PMI should be no more than 72 h for analysis of neuroinflammatory cells to ensure optimal detection and given that none of the cases exceeded 72 h for PMI, analysis should remain reliable.^53^ Further, despite differences in PMI, groups did not differ in GFAP counts (as described below) suggesting that PMI did not alter protein content.

No changes in astrocyte numbers, staining intensity of GFAP, or cell area were detected across all brain regions examined and groups. This finding may be unexpected given that it has been previously shown that AUD reduces the size and number of astrocytes in post‐mortem tissue compared to control subjects,^54^, ^55^, ^56^ and exposure of human cell cultures to alcohol reduces proliferation of astrocytes.^26^ However, the brain regions examined in these studies differed to the current study and were instead focused on the dorsolateral prefrontal cortex, orbitofrontal cortex and hippocampus,^54^, ^55^, ^56^ and thus, like microglia (described above), astrocytes could demonstrate region‐specific responses to alcohol. One possible source of these differences could be astrocyte densities which are known to differ in functions across brains regions.^57^, ^58^ Indeed, studies that have observed decrease in astrocytes in AUD are largely focused in brain regions where there are lower densities of astrocytes,^55^, ^56^, ^59^ while the structures examined in the current study may have high densities of astrocytes; however, further investigation into astrocyte populations in the examined structures is needed. Importantly, it has been demonstrated that GFAP density does not change in rats chronically exposed to alcohol in the cerebellum.^60^ Thus, it may be that alcohol impacts the brain in a region‐specific manner and that GFAP in the putamen, GPe, and VP is not altered in AUD. Additionally, while GFAP is a commonly used and well‐established marker for astrocytes, GFAP has been found to poorly label protoplasmic astrocytes, which are expressed in the examined brain regions of this study.^61^, ^62^ Therefore, there is a need to explore the effect of AUD on astrocytes in human post‐mortem tissue using markers such as Aldh1L1, which reliably labels both protoplasmic and fibrous astrocytes, as well as striatum astrocytes.^61^, ^63^, ^64^ It is important to note that PMI for AUD and remission groups were significantly different, although matched controls; however, at extended PMI it has been shown that GFAP immunoreactivity remains unchanged and astrocytes maintain their morphology.^65^

## 5 CONCLUSION

In summary, the present study adds to the growing body of literature investigating changes in glial cells associated with AUD. In line with prior investigations using animal models of alcohol consumption and human post‐mortem AUD brains, our findings identified an increase in microglia activity in AUD within the putamen, GPe and VP, or alternatively a dystrophic phenotype indicative of damage to microglia. Due to the role that these brain regions play in the development and maintenance of alcohol by controlling habitual learning, motivation, and reward, increased microglia activity may induce changes in normal neuronal functioning in these brain regions to perpetuate alcohol‐seeking behaviours. Moreover, abstinence from alcohol led to the normalisation of microglia activity to levels similar to that of the controls. This suggests that recovery of microglia following abstinence may potentially reverse changes that occurred during periods of alcohol use, although this effect needs to be further investigated given differences in PMI between these groups. In contrast, analysis of astrocytes revealed no significant difference in activity across all groups and brain structures examined suggesting that astrocytes within these regions may not contribute to AUD neuropathology or alcohol‐seeking behaviours. Overall, the findings from this study further characterise the complex pathology of AUD and implicates microglia in the putamen, GPe, and VP as a potential target for treatment for AUD. Thus, emerging microglia‐relevant treatments such as avermectins, minocycline and phosphodiesterase inhibitors may aid in improving AUD.^36^

Case characteristics of subjects including patient ID, age, sex (male (M), female (F)), post‐mortem interval (PMI), brain pH, clinical cause of death (COD) (including chronic obstructive pulmonary disease [COPD]), and years abstinent. ^a^Cases that were excluded from study. ^b^Cases with comorbidities: R40 comorbidity with Parkinson's disease; R10 comorbidity with schizophrenia; R30 comorbidity with benzodiazepine dependence.

Data presented as mean values ± SEM. Control (*n* = 13), AUD (*n* = 13) and Remission (*n* = 9). Male (M); Female (F); Post‐mortem interval (PMI). There was no difference between groups for sex, age, and brain pH. There was also no difference in PMI between AUD and controls and between remission and controls, but there was a difference in between AUD and remission groups.

## CONFLICT OF INTEREST STATEMENT

Authors declare no conflict of interest.

## ETHICS STATEMENT

All experiments were conducted under approval ethics approval (HC200756).

## Supporting information


**Figure S1.** Representative images of morphological analysis of microglia. **B** exhibits the same Iba‐1^+^ cells observed in **A,** with positive pixels highlighted by a yellow outline. These pixels are identified through the ‘Positive Pixel Count’ function in Qupath, with intensity thresholds manually chosen and adjusted for each ROI. Microglia cell bodies are detected using the ‘Positive cell detection’ function on Qupath, as shown in red in **C**. The average area of these red‐highlighted cell bodies in **C** were subsequently subtracted from the average area of each cell, highlighted in yellow in **B**.

## Data Availability

The data that support the findings of this study are available from the corresponding author upon reasonable request.
