# Supplementary figures and images for: Microglia activity in the human basal ganglia is altered in alcohol use disorder and reversed with remission from alcohol
Source: Addict Biol. 2024 Feb 8;29(2):e13374. doi: 10.1111/adb.13374 (PMC10898843; doi:10.1111/adb.13374)

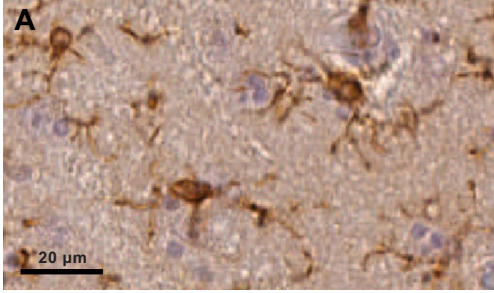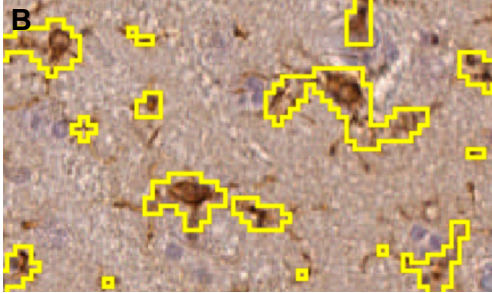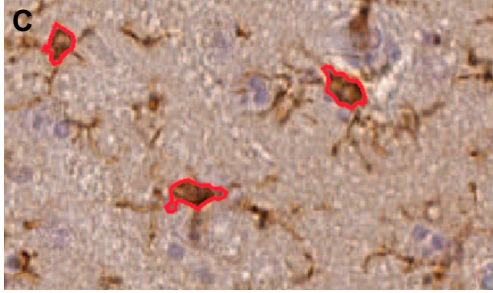

Supplement: Supplementary file 1 — Figure S1. Representative images of morphological analysis of microglia. B exhibits the same Iba‐1+ cells observed in A, with positive pixels highlighted by a yellow outline. These pixels are identified through the ‘Positive Pixel Count’ function in Qupath, with intensity thresholds manually chosen and adjusted for each ROI. Microglia cell bodies are detected using the ‘Positive cell detection’ function on Qupath, as shown in red in C. The average area of these red‐highlighted cell bodies in C were subsequently subtracted from the average area of each cell, highlighted in yellow in B. [file ADB-29-e13374-s001.pdf]
